# Cell Wall Integrity and Its Industrial Applications in Filamentous Fungi

**DOI:** 10.3390/jof8050435

**Published:** 2022-04-23

**Authors:** Akira Yoshimi, Ken Miyazawa, Moriyuki Kawauchi, Keietsu Abe

**Affiliations:** 1 Laboratory of Environmental Interface Technology of Filamentous Fungi, Graduate School of Agriculture, Kyoto University, Kyoto 606-8502, Japan; yoshimi.akira.8c@kyoto-u.ac.jp (A.Y.); kawauchi.moriyuki.8c@kyoto-u.ac.jp (M.K.); 2 ABE-Project, New Industry Creation Hatchery Center, Tohoku University, Sendai 980-8579, Japan; 3 Laboratory of Filamentous Mycoses, Department of Fungal Infection, National Institute of Infectious Diseases, Tokyo 162-8640, Japan; k-miyazawa@niid.go.jp; 4 Laboratory of Applied Microbiology, Graduate School of Agricultural Science, Tohoku University, Sendai 980-8572, Japan

**Keywords:** filamentous fungi, cell wall integrity, signaling pathway, surface sensor, protein kinase C, mitogen-activated protein kinase, plant pathogen, application, fungicide, drug target, culture, productivity

## Abstract

Signal transduction pathways regulating cell wall integrity (CWI) in filamentous fungi have been studied taking into account findings in budding yeast, and much knowledge has been accumulated in recent years. Given that the cell wall is essential for viability in fungi, its architecture has been analyzed in relation to virulence, especially in filamentous fungal pathogens of plants and humans. Although research on CWI signaling in individual fungal species has progressed, an integrated understanding of CWI signaling in diverse fungi has not yet been achieved. For example, the variety of sensor proteins and their functional differences among different fungal species have been described, but the understanding of their general and species-specific biological functions is limited. Our long-term research interest is CWI signaling in filamentous fungi. Here, we outline CWI signaling in these fungi, from sensor proteins required for the recognition of environmental changes to the regulation of cell wall polysaccharide synthesis genes. We discuss the similarities and differences between the functions of CWI signaling factors in filamentous fungi and in budding yeast. We also describe the latest findings on industrial applications, including those derived from studies on CWI signaling: the development of antifungal agents and the development of highly productive strains of filamentous fungi with modified cell surface characteristics by controlling cell wall biogenesis.

## 1. Introduction

Many microorganisms, especially fungi, have evolved as decomposers of terrestrial plants, which are primary producers. Fungi are considered to be among the most successful taxa in terrestrial ecosystems. The success of fungi is thought to be due to their ability to form filamentous cells, called hyphae, which form a network called mycelium; this ability allows fungi to invade solid substrates and acquire nutrients efficiently from the inside of the substrates that are difficult to penetrate for unicellular microorganisms [1,2,3]. The invasion of a solid substrate by filamentous fungi begins with contact between substrate surface and the fungi. When filamentous fungi invade solid substrates, their cells are exposed to oxidative stress; changes in osmolality, temperature, and pH; and chemical compounds, including pheromones [4]. Therefore, biochemical reactions at the cell surface affect fungal growth, and fungal cell surface structures, which form the interface between substrates and fungi, play an important role. Understanding the structure of the fungal cell wall and the regulation of its construction may lead to applications in controlling fungal pathogens and the effective utilization of filamentous fungi.

In this review, we focus on the cell wall integrity (CWI) signaling that regulates cell wall construction and remodeling. The cell wall, the outermost layer of the fungal cell, maintains cell morphology, protects the cells, and transmits the external stimuli inside the cell. Fungal CWI signaling has been studied in detail in the budding yeast *Saccharomyces cerevisiae* (reviewed by Levin [5,6], Gustin et al. [7], and Chen and Thomer [8]). In the CWI pathway of *S.* *cerevisiae*, perturbations of the cell wall are detected by the Wsc-type and Mid-type cell surface sensors. The signal is then consecutively transmitted through the following components: the GDP/GTP exchange factor Rom, the small GTPase Rho1, protein kinase C (PKC), the mitogen-activated protein (MAP) kinase cascade (MAP kinase kinase kinase Bck1; a pair of MAP kinase kinases Mkk1/Mkk2, and the MAP kinase Mpk1/Slt2), and the transcription factors (TFs) Rlm1 and Swi4, a subunit of the Swi4–Swi6 TF complex. The other signaling pathways in *S. cerevisiae* are the high osmolarity glycerol (HOG) pathway (MAP kinase: Hog1 kinase), filamentous and invasive growth (FG) pathway (Kss1 kinase), and pheromone pathway (Fus3 kinase) [9,10]. Extensive crosstalk between these pathways in *S. cerevisiae* has been documented [11,12]. In this review, we refer to the central pathway involved in CWI via PKC–Mpk1/Slt2 or their orthologs as the CWI PKC pathway. When describing the entire system that contributes to the maintenance of CWI, including not only the CWI PKC pathway but also other signaling pathways, we refer to it as CWI signaling.

Our research in this area has resulted in some industrial applications. Here, we discuss the similarities and differences between the functions of CWI signaling factors in filamentous fungi and in yeast, including cell surface sensors in Section 2 and downstream components in Section 3. We describe the development of antifungal agents based on the analysis of CWI signaling in Section 4, and the development of fungal culture technology using strains with modified cell surface structures in Section 5.

## 2. Cell Surface Sensors of Cell Wall Integrity Signaling Pathway

### 2.1. Wsc- and Mid-Type Sensors

Filamentous fungi grow by invading and decomposing solid substrates [3], and these features are used for solid-state fermentation in industrial applications [13,14]. These processes are initiated by a contact between the substrate surface and the fungal cell surface. Fungi perceive information at the contact surface and transmit it into the cells. Cell surface sensors embedded in the cell wall are important in this process, and in sensing and responding appropriately to environmental stresses. Perturbation of the cell wall may affect fungal survival, so changes in cell wall structure as such must also be sensed. Cell wall sensors in fungi were first studied in *S. cerevisiae* (for detailed reviews, see [15,16]). We provide an overview of sensor proteins in fungi in Table 1. The membrane-spanning sensors of the *S. cerevisiae* CWI PKC pathway consist of two sub-families: Wsc-type sensors (Wsc1–3) and Mid-type sensors (Mid2 and Mtl1). All of them have a transmembrane region and an extracellular region; the latter is rich in serine and threonine residues and is highly *O*-mannosylated. At the N-terminus, only the Wsc type has the Wsc domain (also referred to as the cysteine-rich domain, CRD), but only the Mid type has an *N*-glycosylated asparagine residue. The glycan chains of the extracellular region of the sensor proteins are interact with cell wall polysaccharides. These proteins function as mechanosensors. Stimuli in the cell wall and the resulting distortion of the plasma membrane are sensed as force that tilts and stretches the serine/threonine-rich region, which acts like a nanospring [15,16]. This structural change results in a conformational change in the cytoplasmic tail, which triggers downstream signal transmission.

The dimorphic fungus *Candida albicans* forms so-called invasive filaments during host invasion. Strains lacking Wsc-type sensors show little change in susceptibility to cell wall stresses, and the formation of invasive filaments does not differ from that of the wild-type strain [17]. These data suggest that the Wsc-type sensors are not crucial for CWI in this fungus. 

In filamentous fungi, homologs of the *S. cerevisiae* Wsc1–3 cell wall sensors were identified in silico in a model filamentous fungus *Aspergillus nidulans* [18], and their function was analyzed [19,20]. WscA has a Wsc-domain, a serine- and threonine-rich region, a transmembrane region, and a C-terminal intracellular domain. WscA was considered to be a substrate for *O*-d-mannosyltransferase Pmt because Wsc1 and Mid2 are mannosylated by Pmt; this was confirmed using an HA-tagged WscA-expressing strain [19]. Futagami et al. [20] showed that WscA and WscB, both Wsc1 orthologs in *A. nidulans*, are *N*- and *O*-glycosylated and are localized in the cell wall. Disruption of *wscA* results in abnormal growth and reduced conidiation. The conidial formation is also reduced in the *wscB* deletion strain, but to a lesser extent. The *wscAwscB* double-disruption strain is viable, but its growth retardation is more severe than that caused by *wscA* single deletion [20]. Whereas yeast Wsc1 is involved in stress response under alkaline conditions [21], Wsc-type sensors of *A. nidulans* are thought to sense cell wall changes under acidic conditions [20]. Loss of WscA alters the transcript levels of genes for cell wall α-1,3-glucan synthases (*agsA* and *agsB*), resulting in an increase in the content of alkali-soluble glucan [20]. Loss of Wsc-type sensors also enhances the phosphorylation of a mitogen-activated protein (MAP) kinase, MpkA [20]. These results and the absence of α-1,3-glucan in yeast suggest that *A. nidulans* Wsc-type sensors have a somewhat different sensing spectrum and downstream signaling pathway from those of *S. cerevisiae* [20]. Futagami et al. [22] showed that a Mid-type sensor protein, MtlA, in *A. nidulans* is highly *O*-glycosylated and localized to the cell surface. Loss of MtlA decreases conidial formation, increases sensitivity to cell wall inhibitors, such as calcofluor white (CFW), congo red (CR), and micafungin, an echinocandin antifungal, and decreases cell wall glucan and chitin content [22]. Thus, the CWI sensor MtlA is important for cell wall stress tolerance and cell wall maintenance in this fungus [22].

The function of Wsc1–3 and the Mid-type sensor MidA has been reported in the human pathogenic fungus *Aspergillus fumigatus* [23]. The disruption of *A. fumigatus wsc1*, a gene for a Wsc1 homolog of *S. cerevisiae*, increases sensitivity to caspofungin, an echinocandin antifungal, and additional disruption of *wsc3* reduces colony growth and conidial formation. Disruption of *midA* alone does not affect colony growth, but disruption of *midA* in the *wsc1wsc3* double-disruption strain results in severe growth retardation and severe reduction of conidial formation [23]. Disruption of *wsc2* does not affect colony growth or conidiation. MidA, but not Wsc1–3, is essential for the tolerance to CFW, CR, and high-temperature stress [23]. The functions of Wsc1, Wsc3, and MidA partly overlap, and they are involved in vegetative growth and conidiation [23].

In *Neurospora crassa*, loss of WSC-1, a homolog of *S. cerevisiae* Wsc1, increases sensitivity to caspofungin and CFW and strongly reduces the formation of aerial hyphae and conidia [24]. The *wsc-2* gene encodes another Wsc-type sensor; the *wsc-2* disruption strain has a phenotype similar to that of the wild type, but with a slightly reduced growth rate and conidial formation [24]. Disruption of *wsc-1* also reduces the basal level of phosphorylation and stress-induced activity of MAK-1, a MAP kinase in the CWI PKC pathway in *N. crassa*. Disruption of *wsc-2* has a negligible effect on MAK-1 activation by cell wall stress. The authors of [24] concluded that WSC-1 and WSC-2 are required for MAK-1 activation in *N*. *crassa* and that both function as cell wall sensors.

The entomopathogen *Beauveria bassiana* has at least nine proteins with a single Wsc-domain [25,26]. Among them, Wsc1A–E are localized in the hyphal cell wall or membrane, and the deletion of each of them increases sensitivity to cell wall perturbation, osmotic stress, oxidative stress, and metal ions, and also delays germination and reduces resistance to UV-B and/or heat stress [25]. None of the deletions have a significant effect on vegetative growth, conidial formation, or virulence [25]. The ninth Wsc sensor, Wsc1I, which contains not only a Wsc domain but also an N-terminal DUF1996 domain (domain of unknown function 1996), is localized to the vacuoles and cell wall/membrane and is involved in sensitivity to osmotic stress, oxidative stress, and cell wall stress compounds [26]. In a *wsc1I* deletion strain, the phosphorylation level of the MAP kinase Hog1 is greatly reduced under osmotic, oxidative, and cell wall stresses, suggesting that Wsc1I senses a variety of cell stresses upstream of the Hog1 pathway [26]. Overall, the data suggest some variations of Wsc- and Mid-type sensors among fungal species. 

### 2.2. Other Types of Cell Surface Sensors

In addition to the Wsc- and Mid-type sensors, several other types of sensor proteins function at the cell surface in filamentous fungi (Table 1). In *N. crassa*, HAM-7 was identified as a factor associated with anastomosis and sexual development [49]. It has a typical signal peptide at the N-terminus and a glycosylphosphatidylinositol (GPI) anchor signal at the C-terminus [24,49]; it is GPI-anchored to the plasma membrane, and the N-terminal extracellular domain is thought to be localized in the cell wall space. The loss of HAM-7 affects vegetative growth, hyphal branching pattern, and the formation of protoperithecia, but not the sensitivity to cell wall stress compounds [24]. Similar to WSC-1, HAM-7 is required for the activation of MAK-1 MAP kinase, and a strain deficient in both WSC-1 and HAM-7 shows severe phenotypic alterations such as compact colonies, poor formation of aerial hyphae, almost no conidiation, defective cell fusion, and no formation of protoperithecia [24]. Since these alterations are the same as those caused by the deficiency of the MAK-1 pathway, WSC-1 and HAM-7 are considered to be the major sensors upstream of the MAK-1 pathway, although their functions might differ [24].

Signaling mucins are anchored to the plasma membrane, are localized in the cell wall space, and function upstream of MAP kinases [50]. Signaling mucins have a typical signal peptide, a highly glycosylated extracellular inhibitory mucin domain, a single transmembrane domain, and a short intracellular tail. The signaling mucin Msb2 of *S. cerevisiae* is an upstream sensor of the FG and HOG pathways and is activated by nutrient starvation and by cleavage of the extracellular domain [51]. In addition to Msb2, the mucin-like protein Hkr1 is present in *S. cerevisiae* [34] and is involved in sensing cell wall damage by zymolyase [52], which degrades the β-1,3-glucan network [52,53]. Although Hkr1 orthologs have been found in *Ashbya gossypii*, which is closely related to *S. cerevisiae*, they have not been found in other filamentous fungi examined, such as *A. fumigatus*, *Fusarium graminearum*, *Magnaporthe* *grisea* (currently *Magnaporthe oryzae*, synonymous to *Pyricularia oryzae*), and *N. crassa* [54]. In *C. albicans*, Msb2 deficiency leads to increased sensitivity to cell wall stresses and loss of invasive phenotypes [17,55]. Together with the data on the Wsc-type sensor-defective strains [17], this finding suggests that the sensing of cell wall changes in this fungus is dependent more on the Msb2 sensor than on the Wsc-type sensors.

In some plant pathogenic fungi, the function of Msb2 homologs has been analyzed in relation to their virulence [43,46,56]. In the soil-borne vascular wilt fungus *Fusarium oxysporum*, loss of Msb2 leads to phenotypic alterations that overlap with those caused by the deficiency of the Fmk1 MAP kinase pathway (ortholog of the FG pathway in *S. cerevisiae*), including defects in penetration of cellophane membranes, adhesion to host plant roots, and virulence to the host plant [43]. Unlike Fmk1 deficiency, *msb2* deletion confers sensitivity to cell wall stress compounds, and this sensitivity is enhanced by a double knock-out of *msb2* and *fmk1* [43]. These observations indicate that Msb2 is involved in invasive growth and infection upstream of Fmk1, and also in the cell wall stress response through a pathway distinct from the CWI PKC pathway [43]. In the rice blast fungus *M. oryzae*, MoMsb2 functions upstream of the Pmk1 MAP kinase pathway as a sensor for hydrophobicity and cutin monomers on the plant surface; MoMsb2 is involved in appressorium formation in cooperation with MoSho1, which is thought to be an osmo-sensor [45]. Appressorium formation and host invasion via Pmk1 activation involve the interaction of MoMsb2 with the small GTPase Ras2, and MoMsb2 function partially overlaps with that of the mucin-like protein Cbp1 [56], which was originally identified as a chitin-binding protein [57] and lacks the mucin and transmembrane domains. As in other fungi, Msb2 of *Botrytis cinerea* functions as a surface sensor upstream of Bmk1 MAP kinase (ortholog of the Kss1 in *S. cerevisiae*) but seems to have little relevance to the CWI PKC pathway [46]. In *A. nidulans*, Msb2 is involved in adhesion and biofilm formation, cell wall stress tolerance, vegetative growth, and conidiation under nutrient deficiency via both the CWI PKC and FG pathways [40]. In *A. fumigatus*, MsbA has a similar function, with a particularly strong effect on the CWI PKC pathway [41]. The deficiency of MsbA in *A. fumigatus* alters host immune responses and increases virulence, which has been attributed to changes in the cell wall structure [41]. Generally, other types of sensors in filamentous fungi are associated with the CWI PKC pathway, but their contribution varies among fungal species.

## 3. Signal Transduction Downstream of Cell Surface Sensors

### 3.1. Rom2 and Rho1

In *S. cerevisiae*, the cytoplasmic tails of Wsc- and Mid-type sensors interact with the guanine nucleotide exchange factor (GEF) Rom2 [15,16]. This interaction activates the small GTPase Rho1 by converting it into the GTP-bound state. Rho1-GTP activates PKC [15,16], and is also required for the activity of β-1,3-glucan synthase Fks1 (reviewed by Wagener et al. [58]).

The function of Rom2 has been reported in pathogenic fungi *A. fumigatus* [59] and *Candida* species [60]. Since the deletion of *rom2* was suggested to be lethal in *A. fumigatus*, a conditional strain was used for analysis [59]. Under *rom2*-suppressive conditions, this strain has a severe growth defect, a complete loss of conidiation, and an increased sensitivity to cell wall inhibitors [59]. In *A. fumigatus*, Rom2 is localized to the hyphal tip and septa, and *rom2* suppression increases basal levels of phosphorylation of MpkA MAP kinase [59]. Co-immunoprecipitation of HA-tagged Rom2 with Rho1 confirmed their interaction [59]. These results suggest that Rom2 is involved in the activation of the CWI PKC pathway by acting between Wsc- and Mid-type sensors and Rho1 [59].

In a human pathogen, *Candida glabrata*, a temperature-sensitive (ts) mutation in the *rom2* gene has been identified during the analysis of essential genes in ts mutant strains [60]. In *C. albicans*, a strain carrying the same ts mutation was generated; it had colony defects because of the lysis phenotype at a temperature shift without osmostabilizer, as in the ts mutant of *C. glabrata* [60]. These data and the fact that a heterozygous mutant (*Rom2*/*rom2*) but not null mutant (*rom2*/*rom2*) was obtained suggest that the *rom2* gene is essential for viability in these *Candida* species and that *Candida* Rom2 is involved in the CWI PKC pathway, in line with its functional similarities with the yeast Rom2 [60].

The function of Rho1 has been analyzed in *Aspergillus* species [61,62,63,64]. In *A. fumigatus*, AfRho1 forms a complex with β-1,3-glucan synthase Fks1 [61] and, together with Rho3, is localized to the hyphal tip under normal growth conditions and seems to control the CWI PKC pathway and the cytoskeleton [62]. Among five subfamilies of small GTPases, the Rho subfamily is most extensively characterized [65]. The industrial fungus *Aspergillus niger* has six Rho GTPases, and RhoA plays a central role in polarity establishment and survival, RhoB and RhoD are important for the CWI PKC pathway, and RhoD is important for septum formation, while RhoC has a minor function [63]. RacA and CftA (Cdc42) also maintain polarity, but RacA seems to contribute more than Cdc42 in *A. niger* [63]. In *A. nidulans*, RhoA is involved in polar growth, branching, and cell wall biogenesis [64]. In *F. oxysporum*, the loss of the *rho1* gene is not lethal but results in severe growth defects with abnormal cell walls; the cell wall alteration is thought to activate immune responses in host plants [66].

The basidiomycete *Ustilago maydis* causes corn smut disease; an Rho1 homolog of *U. maydis* is required for vegetative growth and is associated with cell polarity and cytokinesis, and Rho1 loss results in abnormalities in budding and chitin deposition [67]. In another basidiomycete, the edible mushroom *Grifola frondosa*, loss of Rho1 results in reduced mycelial growth, decreased amount of cell wall polysaccharides, and increased sensitivity to cell wall stress [68].

### 3.2. Protein Kinase C

At the N-terminus, PKC has C1 and C2 cysteine-rich domains, and Rho1 is thought to interact with the C1 domain to regulate the activity of the CWI PKC pathway [69]. Rho1 also binds the N-terminal HR1A domain of PKC, but this binding seems to be involved in a pathway independent of the CWI PKC pathway [70,71]. At the C-terminus, PKC has a serine/threonine kinase domain and a hydrophobic tail with an NFD (Asn-Phe-Asp) motif, the phenylalanine residue of which coordinates the substrate ATP to activate PKC [69]. 

Comparative genomics has been applied to factors associated with the CWI PKC pathway in human pathogenic fungi such as *C. albicans* and *A. fumigatus* and in plant pathogens such as *M. grisea* and *U. maydis* vs. those in *S. cerevisiae* [54]. The CWI PKC pathway seems to be conserved in most fungal species [54,72]. In *C. albicans*, PKC deficiency leads to cell lysis in both the budding and hyphal growth forms that can be ameliorated by osmotic stabilization [73]. 

The function of PKC of filamentous fungi has been extensively studied in aspergilli, in particular in *A. nidulans* (see our detailed review [74]). Loss of *pkcA* in *A. nidulans* is lethal [75,76]. Repression of this gene increases sensitivity to cell wall stress agents such as caspofungin and CFW and leads to abnormal cell wall structure [77,78]. PkcA has pleiotropic effects and regulates mitosis, germination, secondary metabolism, and farnesol-induced cell death [75,76,77,78]. PkcA inhibits apoptosis induction via the MpkA [79]. Expression of a constitutively active PkcA mutant in *A. nidulans* increases transcript levels of several chitin synthase genes (*chsB*, *chsC*, *chsD*, *csmA*, and *csmB*) and the α-1,3-glucan synthase gene *agsB* [80]. These findings indicate that PkcA in *A. nidulans* regulates the transcription of cell wall-related genes, and at least in this fungus, PkcA seems to play a central role in the CWI PKC pathway. In *A. fumigatus*, *pkcA* is thought to be an essential gene, and the analysis using a non-essential mutant of *pkcA* suggests that PkcA functions upstream of the MAP kinase MpkA [81]. In *N. crassa*, PKC is associated with the light-response signaling pathway and is essential for growth [82,83]. 

The loss of PKC is lethal in many filamentous fungi, so there are not many examples of functional analysis of PKC in plant pathogens. In *M. oryzae*, RNAi-based analysis has shown that the repression of *pkc1* causes severe growth retardation and considerably affects the transcription of genes involved in cell wall remodeling, autophagy, signal transduction, and secondary metabolism [84]. Sugahara et al. [85] found that a filamentous fungus-specific PKC inhibitor suppresses hyphal melanization in *M.* *grisea* by suppressing the expression of melanin synthesis-related genes, which are required for pathogenicity of some plant pathogenic fungi [86,87]. Overall, PKC is important in survival and pathogenesis and the development of drugs targeting fungal PKC may be an effective strategy.

### 3.3. MAP Kinase Cascades Involved in Cell Wall Integrity and Their Targets

In *S. cerevisiae*, the MAP kinase kinase kinase Bck1 activates a pair of redundant MAP kinase kinases Mkk1/Mkk2, and they activate the MAP kinase Mpk1/Slt2 [8]. Scaffold protein Spa2 mediates the interaction between the MAP kinase kinases and MAP kinase [8]. Mpk1/Slt2 phosphorylates the transcription factors (TFs) Rlm1 and Swi4, a subunit of the Swi4–Swi6 TF complex. At least 25 CW-related genes, including genes for β-1,3-glucan synthase and chitin synthases, are regulated by Mpk1/Slt2 [88]. Cell wall stress compounds such as CFW, CR, and zymolyase lead to Mpk1/Slt2 activation [89,90,91]. The transcriptional response to CR is almost exclusively dependent on Mpk1/Slt2 and Rlm1 [92], but the response to cell damage caused by zymolyase requires both CWI and HOG pathways [91]. In response to CWI damage, a complex transcriptional response program associated with altering metabolism and remodeling the cell wall is elaborately implemented [92,93]. Activation of the Mpk1/Slt2 pathway also required for stimulation of calcium influx through the plasma membrane Ca^2+^ channels Cch1–Mid1, resulting in calcineurin activation, TF Crz1 dephosphorylation, its nuclear translocation and transcriptional regulation of genes related to adaptation to cell wall and cytoplasmic stresses [8]. Mpk1/Slt2 is also activated by hyperosmotic stress, which is dependent on the activation of the Mid2 sensor and Hog1 MAP kinase [8]. The regulation of cell wall biogenesis in fungi is linked to various aspects of morphological control and stress responses through active crosstalk with other signaling pathways [8,94].

Although not all of the MAPK orthologs of fungal pathogens such as *C. albicans* have been functionally analyzed fully, their position in the pathway seems to reflect the *S. cerevisiae* paradigm [72]. In *C. albicans*, the response of the CaSko1 transcription factor to caspofungin depends on the Psk1 PAK kinase but not on the Hog1 MAP kinase [72]. In contrast to *S. cerevisiae* Ste11, a MAP kinase kinase kinase of the Kss1 pathway, there is no evidence that *C. albicans* Ste11 activates Hog1 [72]. The Cas5 transcription factor also contributes to the transcriptional response to caspofungin and has no ortholog in *S. cerevisiae* [72].

Among MAP kinases of filamentous fungi, the orthologs of the genes of the CWI PKC pathway have been analyzed in *A. nidulans*, and the differences in their functions between *A. nidulans* and *S. cerevisiae* have been discussed [18]. In *A. nidulans*, the loss of the Mpk1/Slt2 ortholog MpkA modulates conidial germination and polar growth and increases sensitivity to cell wall stress compounds such as micafungin and CFW [18]. The most distinctive difference between *A. nidulans* MpkA and *S. cerevisiae* Mpk1/Slt2 is in their target genes. The transcription of most cell wall-related genes is MpkA-independent, whereas transcription of synthase genes for α-1,3-glucan, which is absent in the cell wall of *S. cerevisiae*, depends on the TF RlmA via MpkA [18]. Transcription of *fksA* for β-1,3-glucan synthase and *chsB* for chitin synthase is MpkA-dependent under some cell wall stresses [22,80], but factors involved in the transcriptional regulation of many other cell wall-related genes are largely unknown. 

In *A. nidulans*, MpkB, an ortholog of Kss1 and Fus3 MAP kinases of the FG and pheromone pathways of *S. cerevisiae* and MpkA have the same phosphorylation motif, and MpkB deletion increases sensitivity to micafungin [95]. Similarly, an MpkB-deficient strain of *A. fumigatus* has an increased sensitivity to caspofungin [96], but at least in *A. nidulans*, MpkB is not involved in the transcriptional regulation of cell wall-related genes [95], suggesting that MpkB may be involved in CWI in a different way. The Kss1/Fus3 orthologous pathway has been extensively studied as a virulence-related factor (see Jiang et al. [97] for a concise and systematic review on this and Mpk1/Slt2 pathways). This pathway is important for appressorium formation and invasive growth of the rice blast fungus *M. oryzae*, and orthologous pathways function similarly in many other appressorium-forming plant pathogens. An MpkB ortholog Chk1 in the southern corn leaf blight fungus *Cochliobolus heterostrophus* regulates not only sexual-asexual development and pathogenicity, but also adaptation to oxidative and heavy-metal stresses [98].

The function of the orthologous Mpk1/Slt2 pathway, especially its involvement in virulence, varies among species in plant pathogenic fungi [97]. Mps1 MAP kinase is dispensable for appressorium formation in *M. oryzae*, but the Mpk1/Slt2 orthologs play a pivotal role in the early stages of appressorium formation in *Colletotrichum lagenarium* and *Colletotrichum gloeosporioides*. They are also involved in various growth processes and pathogenicity-related functions in plant pathogenic fungi: the loss of the Mpk1/Slt2 ortholog leads to severe defects in aerial hyphal formation and sporulation in *M. oryzae* and increases formation of aerial hyphae and decreases that of sclerotium in *Sclerotinia sclerotiorum*.

Among basidiomycetes, the Mpk1/Slt2 orthologous pathway has been analyzed in the pathogenic yeast *Cryptococcus neoformans*. Most of the components of the CWI PKC pathway are conserved except for the sensor proteins in comparison with ascomycete fungal pathogens, and the Mpk1 orthologs seem to share common functions related to cell wall biogenesis, heat stress response, and virulence [99,100]. In *C. neoformans*, Pkc1 activity is important in dynamic morphological changes during infection, especially in changes to the large (so-called Titan) cells and capsule formation [100]. In the edible mushroom *Ganoderma lucidum*, the target of rapamycin (TOR) pathway, which plays a central role in cell growth, regulates cell wall synthesis via an Mpk1/Slt2 ortholog, indicating the potential relationship between the TOR and CWI PKC pathway [101]. In this fungus, the Swi6 ortholog appears to function downstream of the Mpk1/Slt2 pathway; Swi6 has two splice variants, and the variant Swi6B appears to be associated with regulation of the CWI PKC pathway [102]. The development of various CWI regulatory mechanisms in different fungi is due to the complex evolution of the CWI pathway to suit their survival strategies. The development of antifungal agents targeting these unique systems may be effective and is described in Section 4. In addition, the unique technology to increase production by controlling the cell surface structure, which has been developed on the basis of the analysis of CWI signaling, is discussed in Section 5. 

## 4. Cell Wall Integrity as a Drug Target for Antifungal Agents

### 4.1. Compounds That Inhibit the Synthesis of Cell Wall Polysaccharides

The development of antifungal drugs is important in medicine for fungal disease treatment and in agriculture for crop protection. Because the fungal cell wall is essential for survival and its architecture is fungus-specific, factors involved in its construction may be effective targets for antifungal agents. The echinocandin class compounds, such as caspofungin, micafungin, and anidulafungin are semisynthetic lipopeptides; they have been widely used for more than 30 years since their development [103]. Echinocandins have superior antifungal activity against *Candida* spp. and *Aspergillus* spp. and are therapeutic agents in particular for esophageal candidiasis, invasive candidiasis, and invasive aspergillosis [103]. They are also active against some other ascomycetes, including *Alternaria* spp. and *Bipolaris* spp., but not against basidiomycete *C. neoformans* or any zygomycetes [103,104]. Echinocandins inhibit the synthesis of β-1,3-glucan, an essential cell wall component in many fungi; for example, they impair the activity of glucan synthases encoded by the *FKS1* and *FKS2* genes in *S. cerevisiae* [103,104]. The emergence of resistant strains has been reported in some *Candida* spp., and an amino acid substitution in Fks1 seems to contribute to the resistance [104]. Because *Aspergillus* spp. growth is not completely suppressed by echinocandins, it is rather difficult to distinguish whether they are resistant or not, but resistant mutants have been generated in the laboratory [105]. Resistant strains have been also isolated in clinical situations, raising concerns about the increase in the incidence of such strains [105].

The nucleoside antibiotics, blasticidin S and polyoxins, are known as forerunners for antibiotics used for agriculture [106]. Blasticidin S inhibits protein synthesis, whereas polyoxins inhibit cell wall synthesis in target fungi [106,107,108]. Polyoxin A was isolated from *Streptomyces cacaoi* in 1965 [109,110] as a new nucleoside compound and was marketed in 1967 [106]. Polyoxins are structurally similar to the substrate for biosynthesis of chitin (UDP-*N*-acetylglucosamine) [107,108,111], which is an essential cell wall component of plant pathogenic fungi [106,107,108]. Nikkomycins are structurally related to polyoxins [111]. Polyoxins and nikkomycins are taken up by the fungi and mimic the substrate of chitin synthase, antagonistically inhibiting cell wall chitin synthesis [106,107,108,111,112].

Some dyes such as CFW and CR are used for laboratory experiments. CFW and CR bind to the fungal cell wall components chitin and glucans [113] and inhibit cell wall synthesis [114]. Their mechanism of action is well summarized by Ram and Klis [114]. Both compounds have two sulfonic acid groups, which are negatively charged under slightly acidic to basic conditions; this makes the compounds soluble and active against fungi [114]. Under these conditions, they cannot pass through the plasma membrane because they each carry two negative charges and are thought to target compounds on the outside of the cell wall [114]. CFW and CR bind to β-linked-glucans in vitro, but CFW preferentially stains chitin in fungal cell wall [114]. Among the cell wall polysaccharides, β-1,3-glucan interacts strongly with CR but not as strongly with CFW in vitro [113]. In *A. nidulans*, the strain with increased cell surface exposure of β-1,3-glucan due to the loss of α-1,3-glucan from the cell wall has increased CR adsorption [115]. In addition, CR adsorbs more on purified β-1,3-glucan or chitin and less on mutan (bacterial α-1,3-glucan) in vitro [115]. Overall, CFW and CR are thought to act by binding to chitin and β-linked-glucan chains, thereby inhibiting the assembly of chitin and β-linked-glucans and weakening the cell wall [114]. Recently, several transcription factors involved in CR sensitivity and CR dynamics in fungal cells have been analyzed [116]. The amorphous cell surface polysaccharide, galactosaminogalactan (GAG), interferes with the uptake of CR into the fungal cell [116]. CR-resistant strains form larger abnormal swollen (“Quasimodo”) cells than the wild-type or CR-sensitive strains [116]. Those cells adsorb more CR, leading to CR removal from the culture media and resulting in the acquisition of CR resistance [116]. CR affects the transcription of the genes related to primary and secondary metabolism and toxin efflux systems, suggesting that damage to the fungal cell wall can cause serious adverse effects for fungal growth [116].

### 4.2. Compounds That Act on the CWI Signaling

Factors involved in fungal signaling systems are often unique to fungi and so may be effective targets for antifungal drugs. For example, dicarboximide fungicides such as iprodione and procymidone, and phenylpyrrole fungicides such as fludioxonil have been used for many years to control crop diseases [117,118,119,120]. These fungicides convert type III histidine kinases to phosphatases, which deactivate the histidine-containing phosphotransfer intermediator Ypd1, resulting in abnormal activation of the downstream HOG pathway, and disturb the fungal osmotic response signaling system [117,118,119]. 

Recently, Beattie and Krysan [121] developed a high-throughput screening system for antifungal agents based on the adenylate kinase (AK) assay. AK released during fungal cell lysis phosphorylates an ADP-containing reagent, and the generated ATP is detected by luciferase. Using this method, the authors found that compound PIK-75 inhibits growth of not only *A. fumigatus* but also *C. neoformans*, and that PIK-75 activity at least in part is due to the loss of CWI. This assay allows cell-wall active compounds to be identified even if their action on the cell wall is indirect [121].

**Figure 1:**
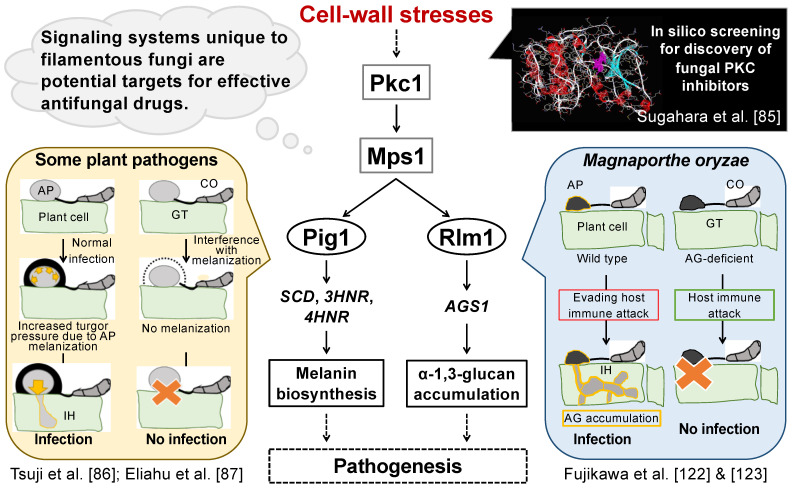
Diagram of drug development to target the CWI signaling pathway in filamentous fungi. In *M. oryzae*, Pkc1 is a protein kinase C, Mps1 is a MAP kinase, and Pig1 and Rlm1 are transcription factors. *SCD* (encoding scytalone dehydratase), *3HNR* (trihydroxy-naphthalene reductase), and *4HNR* (1,3,6,8-tetrahydroxy-naphthalene reductase) are involved in the biosynthesis of 1,8-dihydroxynaphthalene (DHN) melanin in several plant pathogens, including *M. oryzae. AGS1* encodes α-1,3-glucan synthase. Abbreviations: AP, appressorium; GT, germ tube; CO, conidium; IH, invasive hyphae; AG, α-1,3-glucan.

As described in *3.1*, PKC is an important signaling factor associated with cell wall biogenesis. Therefore, PKC inhibitors, such as staurosporine, enzastaurin, and ruboxistaurin, can be expected to have excellent antifungal activity. For example, staurosporine strongly inhibits PKC in filamentous fungi [85]. However, these inhibitors cannot be used as antifungal agents because they also inhibit human PKC. To screen for specific inhibitors of fungal cell wall biogenesis, Sugahara et al. [85] conducted an in silico screening to target PKC of *M. oryzae*. The overall concept of the study is depicted in Figure 1. A three-dimensional MgPkc1 structure was modeled to screen for compounds that might inhibit its kinase domain, and the candidate compounds were tested for antifungal activity against *M. grisea.* Among them, Z-705 had the highest inhibitory effect. Chimeric *PKCs* encoding the regulator domain from *S. cerevisiae* and the kinase domain from *S. cerevisiae* (control), *M. grisea* or *A. nidulans* were integrated in the *S. cerevisiae* genome, and Z-705 specifically inhibited chimeric PKCs with the kinase domain from filamentous fungi, but not with that from *S. cerevisiae* [85]. The inhibitory effect was comparable to that of staurosporine, a well-known PKC inhibitor. This compound also inhibited hyphal melanization induced by cell wall stress in *M. grisea*, which is necessary for the infection process [86,87] (Figure 1). In *M. grisea*, Mps1 acts downstream of Pkc1 and regulates gene expression for synthase of α-1,3-glucan that confers the ability to evade the host plant immune system [122,123]. From this perspective, Pkc1 inhibition is also important for reducing pathogenicity. We believe that the efficacy of drugs targeting the cell wall of filamentous fungi is increasing, and the development of such drugs should be further promoted in the future.

## 5. Improvement of Productivity by Modification of Macromorphology in Filamentous Fungi

Another applied approach derived from studies on cell wall biogenesis in filamentous fungi is the improvement of culture characteristics to increase productivity by controlling the surface properties of fungal cells and fungal morphology. Here, we describe some examples of this approach.

### 5.1. Phenotypes of α-1,3-Glucan-Deficient Mutants

As described above, expression of α-1,3-glucan synthase genes (*agsA*, *agsB*) is controlled by MAP kinase MpkA in *A. nidulans* [18]. Single or double disruption of the two α-1,3-glucan synthase genes of *A. nidulans* has revealed that the single disruption of *agsB* and the double disruption of *agsA* and *agsB* cause complete loss of cell wall α-1,3-glucan but are not lethal [115]. *Aspergillus fumigatus* has three α-1,3-glucan synthase genes, and disruption of all the three genes (Δ*ags*) is not lethal [124,125]. The germinating conidia of *A*. *fumigatus* Δ*ags* do not aggregate [124]. The hyphae of α-1,3-glucan-deficient mutants of *A. nidulans* such as Δ*agsB* and Δ*agsA*Δ*agsB* are dispersed in shake-flask cultures, whereas those of the parental strain form tightly aggregated pellets [18]. In *A. nidulans*, the *agsB* gene is clustered with the α-amylase-encoding genes *amyD* and *amyG* [126]. An intracellular α-amylase AmyG hypothetically contributes to synthesis of the primer molecule for α-1,3-glucan polymerization by the α-1,3-glucan synthase [126,127]. Disruption of the *amyG* gene results in a substantial decrease in the content of cell wall α-1,3-glucan and lead to hyphal dispersion or formation of tiny hyphal pellets [126,127]. Disruption or overexpression of *amyD*, which encodes GPI-anchored α-amylase, increases or decreases cell wall α-1,3-glucan, respectively [126]. The hyphae of the *amyD* overexpression strain show a phenotype similar to those of the α-1,3-glucan-deficient mutants [126,128]. These results suggest that AmyD represses α-1,3-glucan biosynthesis. Overexpression of AmyD without the C-terminal GPI-anchor in *A. nidulans* scarcely affects cell wall α-1,3-glucan, suggesting the importance of the GPI anchor for correct cellular localization and function of AmyD [129]. In *A. fumigatus*, treatment with α-1,3-glucanase removes α-1,3-glucan from conidia and leads to their dispersion in medium, indicating the involvement of α-1,3-glucan in conidial aggregation [130]. Dispersion of hyphae in *A. nidulans* in α-1,3-glucan-deficient mutants and that of germinating conidia in *A. fumigatus* α-1,3-glucan-deficient mutants suggests that α-1,3-glucan functions as an aggregation factor for hyphae and conidia. Interestingly, *A. nidulans* Δ*agsB* and Δ*agsA*Δ*agsB* mutants produce considerably more hyphal cells than the wild-type strain does under submerged culture conditions, implying that the dispersed hyphal cells of the α-1,3-glucan deficient mutants can be used for fermentation of valuable products. The α-1,3-glucan deficient mutants of *A. nidulans* show better production of endogenous penicillin and α-amylase than the wild type [131].

In the α-1,3-glucan-deficient mutant of the industrial fungus *A. oryzae*, three α-1,3-glucan synthase genes are disrupted [132]. The *A. oryzae* Δ*agsA*Δ*agsB*Δ*agsC* mutant forms smaller hyphal pellets than the parental wild-type strain, suggesting that α-1,3-glucan is also an aggregation factor in *A. oryzae*. This mutant produces more recombinant protein than the wild-type strain [133]. Jeennor et al. [134] reported that the disruption of the *ags1* gene (probably *agsB* in Miyazawa’s work [133]) in *A. oryzae* significantly improves lipid production in a stirred-tank bioreactor. The disruptant of the *Aspergillus luchuensis agsE* gene, an ortholog to *A. nidulans agsB*, shows better protoplast formation than the wild-type strain when treated with the cell wall lytic enzyme Yatalase [135]. The *A. fumigatus* mutants in which the *ags1* gene, an ortholog of *A. nidulans agsB*, is disrupted, form smaller hyphal pellets than the wild type [136]. Taken together, α-1,3-glucan is an aggregation factor for hyphae and conidia in *Aspergillus* fungi.

### 5.2. Phenotypes of Galactosaminogalactan-Deficient Mutants

In *Aspergillus* species, GAG is one of the components of the extracellular matrix and is essential for biofilm formation [137]. In the background of the defect of α-1,3-glucan biosynthesis (Δ*agsA*Δ*agsB*Δ*agsC*) in *A. oryzae*, disruption of the *sphZ* and *ugeZ* genes (AGΔ-GAGΔ), which are speculative GAG biosynthetic genes of *A. oryzae*, leads to dispersion of hyphae under submerged culture conditions, suggesting that GAG also contributes to aggregation in *A. oryzae* [138]. A simultaneous defect of α-1,3-glucan and GAG biosynthesis also leads to hyphal dispersion in *A. fumigatus* [136]. In *B. cinerea* and *Cochlioborus heterostrophus*, GAG also contributes to hyphal aggregation [139]. Recently, Mei et al. [140] reported that the insect pathogenic fungus *Metarhizium robertsii* has GAG biosynthetic genes, and defects of GAG biosynthesis lead to hyphal dispersion. In ascomycetes, GAG biosynthetic genes are found in some *Pezizomycotina*, and only in *Trichosporon asahii* in basidiomycetes [141]. Expression of GAG biosynthetic genes is thought to be regulated by transcription factors such as StuA, MedA, and SomA in *A. fumigatus* [142,143,144]. Since disruption of the *agdZ* gene increases GAG secretion in *A. oryzae* and *A. fumigatus* (Miyazawa et al., unpublished results), some mechanisms might sense adhesion of hyphae and subsequently downregulate GAG biosynthesis.

### 5.3. Improvement of Productivity Using a Mutant Lacking both α-1,3-Glucan and GAG

**Figure 2:**
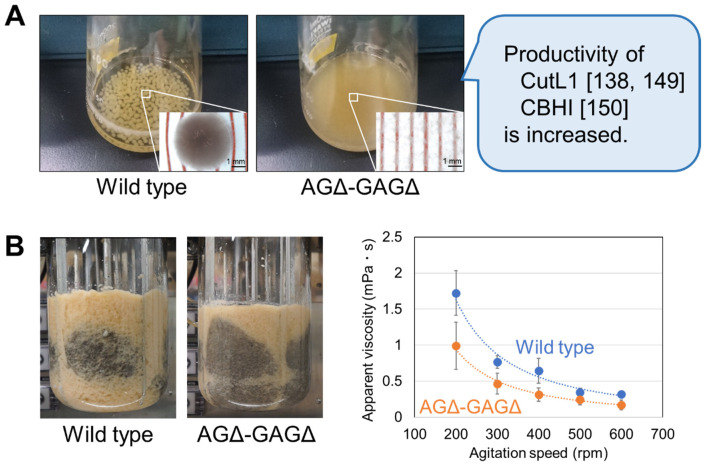
Improvement of productivity with the *Aspergillus oryzae* mutant lacking both α-1,3-glucan and GAG (AGΔ-GAGΔ). (**A**) Growth of the wild-type and AGΔ-GAGΔ strains in liquid culture. Although the wild type forms pellets of several millimeters, the AGΔ-GAGΔ hyphae are fully dispersed. This unique macromorphology of AGΔ-GAGΔ results in increased production of secreted recombinant polyesterase CutL1 and recombinant cellulase CBHI. Conidia (1.0 × 10^5^/mL) of each strain were inoculated into 50 mL of YPD (2% peptone, 1% yeast extract and 2% glucose) medium in a 200 mL Erlenmeyer flask and rotated at 120 rpm at 30 °C. Magnified images (bottom right) were taken under a stereomicroscope. (**B**) AGΔ-GAGΔ culture has improved rheological properties. The wild type and AGΔ-GAGΔ expressing recombinant *cutL1* gene were cultured in YPDS (6% peptone, 1% yeast extract, 6% glucose and 20 mM succinate buffer) in a 5 L lab-scale bioreactor. Left panels: Chinese ink was dropped onto the culture surface at 60 h, and diffusion was imaged at 6 s. Right panel: Apparent viscosity of the culture at 36 h. Torque values were measured with a mixing torquemeter, and apparent viscosity was calculated from the *Np*-*Re* diagram at the indicated agitation speeds.

Regulation of macromorphology such as hyphal pellets and pulp form has been a key issue in fermentation using filamentous fungi [145]. Macromorphology of filamentous fungi is controlled by adjusting culture conditions such as agitation speed, pH, and medium composition [146,147]. Recently, addition of microparticles such as titanate and talc to liquid culture media has been found to promote the formation of micro-pellets that can improve productivity of fermentation of filamentous fungi [148]. We here illustrate our strategy for improving productivity with cell wall mutants of *A. oryzae* (Figure 2). Miyazawa et al. [138] showed that hyphae of the AGΔ-GAGΔ mutant are fully dispersed under submerged culture conditions, and production of recombinant polyesterase CutL1 is significantly higher in AGΔ-GAGΔ than in the parental wild-type strain in shake-flask culture [138]. Ichikawa et al. [149] showed that the production of secreted CutL1 was higher in AGΔ-GAGΔ than in the wild type or mutants lacking α-1,3-glucan (AGΔ) or GAG (GAGΔ) in batch culture in a 5 L lab-scale bioreactor. The apparent viscosity of the AGΔ-GAGΔ culture tended to be lower than that of the wild-type strain culture at each agitation speed examined (200–600 rpm), suggesting that the lack of α-1,3-glucan and GAG in the hyphae improves culture rheology, increasing recombinant protein production [149]. Sakuragawa et al. [150] reported that the AGΔ-GAGΔ strain produces more recombinant cellulase CBHI than the wild-type strain in a 250 mL bioreactor. The AGΔ-GAGΔ strain shows rapid glucose consumption, increased mycelial dry weight, and higher respiration activity in comparison with the wild-type strain. The levels of metabolites of glycolysis and TCA cycle are lower in AGΔ-GAGΔ than in the wild type in liquid culture, suggesting that AGΔ-GAGΔ shows higher metabolic flux than the wild type [150]. Since the production of beneficial compounds from fungal cells is attributable to complex physiological events, the mechanisms underlying the productivity of AGΔ-GAGΔ in the bioreactor are presently being analyzed. Further improvement of the productivity is expected to be achieved by conferring stress susceptibility to the AGΔ-GAGΔ mutant and fine tuning the culture conditions through the screening for stress factors and multi-omics analyses in the cultivation.

### 5.4. Improvement of Productivity by Mutations in Cell Wall-Related Genes

Both extracellular hydrolytic enzymes such as amylases and proteases and cell wall synthesizing enzymes are packaged in vesicles and delivered from the Golgi to the hyphal tip of filamentous fungi [151]. Delivery of cell wall synthesizing enzymes to the hyphal tip balances necessity to secrete extracellular enzymes for nutrient acquisition [151]. Since secretion of enzymes and cell wall biogenesis are linked, perturbation to cell wall biogenesis seems to considerably affect enzyme secretion [151]. 

The *A. niger* SH2 strain is widely used in industrial enzyme production [151,152]. In the SH2 genome sequence, Yin et al. [152] found frame-shift mutations and non-synonymous SNPs in genes of CWI signaling, β-1,3-glucan synthesis and chitin synthesis and suggested that they affect hyphal development and hyphal fragmentation during industrial fermentation. Sun et al. [153] constructed *A. niger* mutants with the silenced chitin synthase gene *chsC*. The mutants showed shorter hyphae with lower proportion of dispersed mycelia, decreased viscosity and improved oxygen and mass transfer efficiency, which consequently improved production of citric acid [153]. Yin et al. [154] evaluated citrate production by *A. niger* H915-1 (an industrial producer) and by A1 and L2 (“degenerated” isolates of H915-1) strains. The H915-1 forms bulbous hyphae with short, swollen branches during citrate fermentation, and has the highest citrate titer, whereas A1 forms fewer compact pellets and L2 forms mycelial clumps [154]. Yin et al. [154] indicated that these differences in morphology may influence medium viscosity and hyphal respiration [154]. For citrate generation, the tight pellet form but not the diffuse filamentous form is preferred [154]. Liu et al. [155] reported that silencing of the *chs4* gene encoding class III chitin synthase in *Penicillium chrysogenum* by RNA interference causes formation of a smaller pellet, hyper-branched hyphae, and improves penicillin production. To find *N. crassa* mutants with decreased viscosity in submerged culture, Lin et al. [156] screened 90 morphological mutants and found two such mutants. The causing gene *gul*-*1* encodes an mRNA-binding protein. Disruption of this gene downregulates GPI-anchored cell wall proteins, upregulates non-GPI cell wall proteins, and alters expression of the hydrophobin gene. Disruption of *gul*-*1* in the hyper-cellulase–producing strain significantly decreases culture viscosity compared to the parental strain. Fiedler et al. [157] analyzed the transcriptomics of *A. niger* cells treated with inhibitors of synthesis of chitin (CFW), glucan (caspofungin), sphingolipids (aureobasidin A), and ergosterol (fenpropimorph), and of calcium/calcineurin signaling (FK506), which directly or indirectly interfere with CWI. The analysis suggests that (i) the CWI PKC pathway as a main compensatory response is induced by caspofungin via RhoB and by aureobasidin A via RhoD, followed by activation of the MAPKK MkkA and the TF RlmA; (ii) RlmA is the main TF for protection against CFW, but it cooperates with MsnA and CrzA for protection against caspofungin and aureobasidin A; (iii) aureobasidin A, but not fenpropimorph, induces cell wall stress.

Overall, the macromorphology of filamentous fungi closely relates to productivity. Although several components regulated by the CWI PKC pathway in the production strains have been revealed, how to regulate the CWI PKC pathway to improve productivity is scarcely understood. Combining the screening of phenotypic mutants and analysis of the mechanisms underlying cellular physiology as described by Lin et al. [156] could lead to a breakthrough technology to further improve fungal productivity.

## 6. Conclusions and Perspectives

The cell wall of filamentous fungi is constantly exposed to the environment and is closely involved in interactions with other microorganisms, plants and animals. The fungal cell wall, as well as those of bacteria and plants, is mainly composed of polysaccharides, but these polysaccharides and their structures are quite different from those of bacteria and plants. Although the PKC is conserved in all eukaryotes, CWI PKC pathway has evolved independently in fungi and varies even at the species level. Perturbing CWI signaling is an effective strategy for controlling fungal growth. Chemical compounds that target certain signaling factors of CWI signaling can be used to control pathogens of plants and animals. Effective antifungal drugs targeting the cell wall biosynthesis of filamentous fungi are now on the market, and the screening for and consequent development of such chemicals are underway. Since the genomic information of filamentous fungi is continuously accumulated and artificial intelligence (AI)-based analyses are advancing in various fields, the development of antifungal drugs targeting CWI signaling will be further accelerated by utilizing AI technology in the analysis of genomic information.

The studies of CWI signaling have revealed that polysaccharides such as α-1,3-glucan and GAG function as adhesive factors for hyphae in aspergilli and cause the formation of hyphal pellets. Regulation of the display of these polysaccharides on the cell surface enables filamentous fungi to control their macromorphology such as pellets and pulp forms. Filamentous fungi are extensively used for large-scale industrial cultivation in submerged culture for production of proteins and low-molecular-weight chemicals. However, the capacity of production by filamentous fungi does not reach that by the unicellular fungus *S. cerevisiae* or bacteria *Escherichia coli* and *Bacillus subtilis*, because of the unstable macromorphology of filamentous fungi during liquid cultivation. Several attempts have been made to control the hyphal morphology in filamentous fungi to improve the cultivation characteristics, but the fundamental technology to control hyphal pellet formation has not been established. Modifying polysaccharide contents of the cell surface has led to strains with dispersed hyphae and normal growth, which ensures the efficient acquisition of nutrients and dissolved oxygen. Further analysis of the mechanisms of cell wall biogenesis in filamentous fungi will generate knowledge that will lead to the development of antifungal agents and may also lead to innovative technology for industrial cultivation using filamentous fungi. Therefore, studies on the cell wall biogenesis of filamentous fungi should be continuously promoted, so that the ensuing fruitful achievements can contribute to the improvement of human life.

## Figures and Tables

**Table 1:** Major fungal surface sensors whose functions have been analyzed.

Phylum	Subphylum	Class	Species	Sensor Name	Type	Typical Phenotype(s) of Deficient Strain in Relation to CWI *	References
Ascomycota	Taphrinomycotina	Schizosaccharomycetes	*Schizosaccharomyces pombe*	Wsc1	Wsc	Slightly sensitive to CFG.	[27]
				Mtl2	Mid	Sensitive to CFG, CAF, vanadate, NaCl, H_2_O_2_, and SDS. Decreased β-1,3-glucan content in CW.	[27]
	Saccharomycotina	Saccharomycetes	*Saccharomyces cerevisiae*	WSC1	Wsc	Cell lysis defect and thermosensitive growth defect at 37 °C on YPD medium.	[28,29,30]
				WSC2	Wsc	Deletion of *WSC2* and/or *WSC3* exacerbates the phenotype of the *wsc1*∆ strain.	[29]
				WSC3	Wsc	Deletion of *WSC2* and/or *WSC3* exacerbates the phenotype of the *wsc1*∆ strain.	[29]
				WSC4	Wsc-like	Not generated.	[29,31]
				Mid2	Mid	Resistant to CFW. Changes in growth rate and viability in a number of different cell wall biosynthesis mutants.	[32,33]
				Mtl1	Mid	Not sensitive to thermo-, oxidative, or osmotic stresses or CFW.	[32]
				Msb2	Signaling mucin	Severely osmosensitive in combination with the deficiency in another mucin-like protein, Hkr1.	[34]
			*Candida albicans*	Wsc1	Wsc	Normal resistance to CR and CFW.	[17]
				Wsc2	Wsc	Normal resistance to CR but lower sensitivity to CFW.	[17]
				Msb2	Signaling mucin	Growth defects at 30 °C and 37 °C and a striking growth defect at 42 °C.	[35,36]
			*Pichia pastoris*	PpWsc1	Wsc	Sensitive to high temperature and CR.	[37]
				PpWsc2	Wsc	Not sensitive to high temperature or CR.	[37]
				PpWsc3	Wsc	Not sensitive to high temperature or CR.	[37]
			*Kluyveromyces lactis*	KlWsc1	Wsc	Sensitive to CAF and CR in combination with *KlMid2* disruption.	[38]
				KlWsc2/3	Wsc	Sensitive to CAF and CR in combination with *KlWsc1* and *KlMid2* disruption.	[38]
				KlMid2	Mid	Sensitive to CAF and CR in combination with *KlWsc1* disruption.	[38]
	Pezizomycotina	Eurotiomycetes	*Aspergillus nidulans*	WscA	Wsc	Reduced colony and conidia formation under acidic conditions or not. High frequency of swollen hyphae under hypo-osmotic conditions.	[20]
				WscB	Wsc	Reduced conidiation and growth inhibition under acidic conditions, but to a lesser extent than those caused by a WscA defect.	[20]
				MtlA	Mid	Reduced conidiation. Growth deficiency in the presence of CW inhibitor. Reduction in the glucan and chitin contents in CW.	[22,39]
				MsbA	Signaling mucin	Sensitive to CR, CFW, and cation stresses (MnCl_2_).	[40]
			*Aspergillus fumigatus*	Wsc1	Wsc	Less dense at the colony fringe, but only a mardinal decrease in radial growth. Increased sensitivity to CFG.	[23]
				Wsc2	Wsc	No effect of disruption even in the ∆*wsc1* background.	[23]
				Wsc3	Wsc	Impaired radial growth and reduced conidiation in the ∆*wsc1* background.	[23]
				MidA	Mid	Highly sensitive to CR, CFW, and elevated temperature.	[23]
				MsbA	Signaling mucin	Impaired radial growth. Significant delay in germ tube formation. Sensitive to CR, CFW, nikkomycin Z, and NaCl.	[41]
		Sordariomycetes	*Neurospora crassa*	WSC-1	Wsc	Compact growth. Poor aerial hyphae formation. Almost aconidial. Sensitive to CFG and CFW.	[24]
				WSC-2	Wsc	Slightly reduced growth rate and conidiation.	[24]
				HAM-7	Other	Altered growth and branching pattern. Reduced aerial hyphal formation. No protoperithecia. Defective in cell fusion.	[24]
			*Fusarium graminearum*	Wsc2B	Wsc	Defects in hyphal growth, virulence, and response to CW stresses (cellulase, lysozyme, and snailase).	[42]
			*Fusarium oxysporum*	Msb2	Signaling mucin	Significantly slower growth on low-nitrogen medium but not on nutrient-rich medium. Sensitive to CR and CFW.	[43]
			*Beauveria bassiana*	Wsc1A	Wsc	Increased sensitivity to CW stress, oxidation, high osmolarity. No effect on growth, conidiation, or virulence.	[25]
				Wsc1B	Wsc	Increased sensitivity to CW stress, oxidation, high osmolarity. No effect on growth, conidiation, or virulence.	[25]
				Wsc1C	Wsc	Increased sensitivity to CW stress, oxidation, high osmolarity. No effect on growth, conidiation, or virulence.	[25]
				Wsc1D	Wsc	Increased sensitivity to CW stress, oxidation, high osmolarity. No effect on growth, conidiation, or virulence.	[25]
				Wsc1E	Wsc	Increased sensitivity to CW stress, oxidation, high osmolarity. No effect on growth, conidiation, or virulence.	[25]
				Wsc1I	Wsc-like?	Increased sensitivity to CW stress, oxidation, high osmolarity. No effect on growth, conidiation, or virulence.	[26]
			*Metarhizium rileyi*	MrWsc1	Wsc	Targeted knockout has not been successful.	[44]
				MrMid2	Mid	Impaired dimorphic transition, conidiation, and microsclerotium. Sensitive to thermal, CW, and oxidative stresses. Decreased virulence.	[44]
			*Pyricularia oryzae*	MoMsb2	Signaling mucin	Significantly reduced appressorium formation and virulence. Slightly reduced growth rate.	[45]
		Leotiomycetes	*Botrytis cinerea*	Msb2	Signaling mucin	Normal growth. Almost unable to form appressoria or infection cushions on hard surfaces.	[46]
Basidiomycota	Ustilaginomycotina	Ustilaginomycetes	*Ustilago maydis*	Msb2	Signaling mucin	Impaired host colonization and appressorium formation on plant surface.	[47]
	Agaricomycotina	Tremellomycetes	*Cryptococcus neoformans*	Msb2	Signaling mucin	Resistant to osmotic stress. No thermosensitivity but marginally increased sensitivity to cryostress.	[48]

* Abbreviations: CWI, cell wall integrity; CFG, caspofungin; CAF, caffeine; SDS, sodium dodecyl sulfate; CW, cell wall; CR, congo red; CFW, calcofluor white.

## References

[1] Cairns T.C., Zheng X., Zheng P., Sun J., Meyer V. (2021). Turning inside out: Filamentous fungal secretion and its applications in biotechnology, agriculture, and the clinic. J. Fungi.

[2] Treseder K.K., Lennon J.T. (2015). Fungal traits that drive ecosystem dynamics on land. Microbiol. Mol. Biol. Rev..

[3] Gadd G.M. (2017). The geomycology of elemental cycling and transformations in the environment. Microbiol. Spectr..

[4] Hagiwara D., Yoshimi A., Sakamoto K., Gomi K., Abe K., Takagi H., Kitagaki H. (2015). Response and adaptation to cell wall stress and osmotic stress in *Aspergillus* species. Stress Biology of Yeasts and Fungi: Applications for Industrial Brewing and Fermentation.

[5] Levin D.E. (2005). Cell wall integrity signaling in *Saccharomyces cerevisiae*. Microbiol. Mol. Biol. Rev..

[6] Levin D.E. (2011). Regulation of cell wall biogenesis in *Saccharomyces cerevisiae*: The cell wall integrity signaling pathway. Genetics.

[7] Gustin M.C., Albertyn J., Alexander M., Davenport K. (1998). MAP kinase pathways in the yeast *Saccharomyces cerevisiae*. Microbiol. Mol. Biol. Rev..

[8] Chen R.E., Thorner J. (2007). Function and regulation in MAPK signaling pathways: Lessons learned from the yeast *Saccharomyces cerevisiae*. Biochim. Biophys. Acta.

[9] Hohmann S. (2009). Control of high osmolarity signalling in the yeast *Saccharomyces cerevisiae*. FEBS Lett..

[10] Cullen P.J., Sprague G.F. (2012). The regulation of filamentous growth in yeast. Genetics.

[11] McClean M.N., Mody A., Broach J.R., Ramanathan S. (2007). Cross-talk and decision making in MAP kinase pathways. Nat. Genet..

[12] Saito H. (2010). Regulation of cross-talk in yeast MAPK signaling pathways. Curr. Opin. Microbiol..

[13] Abe K., Gomi K., Hasegawa F., Machida M. (2006). Impact of *Aspergillus oryzae* genomics on industrial production of metabolites. Mycopathologia.

[14] Machida M., Yamada O., Gomi K. (2008). Genomics of *Aspergillus oryzae*: Learning from the history of Koji mold and exploration of its future. DNA Res..

[15] Kock C., Dufrene Y.F., Heinisch J.J. (2015). Up against the wall: Is yeast cell wall integrity ensured by mechanosensing in plasma membrane microdomains?. Appl. Environ. Microbiol..

[16] Jendretzki A., Wittland J., Wilk S., Straede A., Heinisch J.J. (2011). How do I begin? Sensing extracellular stress to maintain yeast cell wall integrity. Eur. J. Cell Biol..

[17] Zucchi P.C., Davis T.R., Kumamoto C.A. (2010). A *Candida albicans* cell wall-linked protein promotes invasive filamentation into semi-solid medium. Mol. Microbiol..

[18] Fujioka T., Mizutani O., Furukawa K., Sato N., Yoshimi A., Yamagata Y., Nakajima T., Abe K. (2007). MpkA-dependent and -independent cell wall integrity signaling in *Aspergillus nidulans*. Eukaryot. Cell.

[19] Goto M., Harada Y., Oka T., Matsumoto S., Takegawa K., Furukawa K. (2009). Protein *O*-mannosyltransferases B and C support hyphal development and differentiation in *Aspergillus nidulans*. Eukaryot. Cell.

[20] Futagami T., Nakao S., Kido Y., Oka T., Kajiwara Y., Takashita H., Omori T., Furukawa K., Goto M. (2011). Putative stress sensors WscA and WscB are involved in hypo-osmotic and acidic pH stress tolerance in *Aspergillus nidulans*. Eukaryot. Cell.

[21] Serrano R., Martin H., Casamayor A., Arino J. (2006). Signaling alkaline pH stress in the yeast *Saccharomyces cerevisiae* through the Wsc1 cell surface sensor and the Slt2 MAPK pathway. J. Biol. Chem..

[22] Futagami T., Seto K., Kajiwara Y., Takashita H., Omori T., Takegawa K., Goto M. (2014). The putative stress sensor protein MtlA is required for conidia formation, cell wall stress tolerance, and cell wall integrity in *Aspergillus nidulans*. Biosci. Biotechnol. Biochem..

[23] Dichtl K., Helmschrott C., Dirr F., Wagener J. (2012). Deciphering cell wall integrity signalling in *Aspergillus fumigatus*: Identification and functional characterization of cell wall stress sensors and relevant Rho GTPases. Mol. Microbiol..

[24] Maddi A., Dettman A., Fu C., Seiler S., Free S.J. (2012). WSC-1 and HAM-7 are MAK-1 MAP kinase pathway sensors required for cell wall integrity and hyphal fusion in *Neurospora crassa*. PLoS ONE.

[25] Tong S.M., Chen Y., Zhu J., Ying S.H., Feng M.G. (2016). Subcellular localization of five singular WSC domain-containing proteins and their roles in *Beauveria bassiana* responses to stress cues and metal ions. Environ. Microbiol. Rep..

[26] Tong S.M., Wang D.Y., Gao B.J., Ying S.H., Feng M.G. (2019). The DUF1996 and WSC domain-containing protein Wsc1I acts as a novel sensor of multiple stress cues in *Beauveria bassiana*. Cell. Microbiol..

[27] Cruz S., Munoz S., Manjon E., Garcia P., Sanchez Y. (2013). The fission yeast cell wall stress sensor-like proteins Mtl2 and Wsc1 act by turning on the GTPase Rho1p but act independently of the cell wall integrity pathway. MicrobiologyOpen.

[28] Gray J.V., Ogas J.P., Kamada Y., Stone M., Levin D.E., Herskowitz I. (1997). A role for the Pkc1 MAP kinase pathway of *Saccharomyces cerevisiae* in bud emergence and identification of a putative upstream regulator. EMBO J..

[29] Verna J., Lodder A., Lee K., Vagts A., Ballester R. (1997). A family of genes required for maintenance of cell wall integrity and for the stress response in *Saccharomyces cerevisiae*. Proc. Natl. Acad. Sci. USA.

[30] Jacoby J.J., Nilius S.M., Heinisch J.J. (1998). A screen for upstream components of the yeast protein kinase C signal transduction pathway identifies the product of the *SLG1* gene. Mol. Gen. Genet..

[31] Wei Y.F., Chen B.J., Samson L. (1995). Suppression of *Escherichia coli alkB* mutants by *Saccharomyces cerevisiae* genes. J. Bacteriol..

[32] Ketela T., Green R., Bussey H. (1999). *Saccharomyces cerevisiae* Mid2p is a potential cell wall stress sensor and upstream activator of the PKC1-MPK1 cell integrity pathway. J. Bacteriol..

[33] Rajavel M., Philip B., Buehrer B.M., Errede B., Levin D.E. (1999). Mid2 is a putative sensor for cell integrity signaling in *Saccharomyces cerevisiae*. Mol. Cell. Biol..

[34] Tatebayashi K., Tanaka K., Yang H.Y., Yamamoto K., Matsushita Y., Tomida T., Imai M., Saito H. (2007). Transmembrane mucins Hkr1 and Msb2 are putative osmosensors in the SHO1 branch of yeast HOG pathway. EMBO J..

[35] Puri S., Kumar R., Chadha S., Tati S., Conti H.R., Hube B., Cullen P.J., Edgerton M. (2012). Secreted aspartic protease cleavage of *Candida albicans* Msb2 activates Cek1 MAPK signaling affecting biofilm formation and oropharyngeal candidiasis. PLoS ONE.

[36] Saraswat D., Kumar R., Pande T., Edgerton M., Cullen P.J. (2016). Signalling mucin Msb2 Regulates adaptation to thermal stress in *Candida albicans*. Mol. Microbiol..

[37] Ohsawa S., Yurimoto H., Sakai Y. (2017). Novel function of Wsc proteins as a methanol-sensing machinery in the yeast *Pichia pastoris*. Mol. Microbiol..

[38] Rodicio R., Buchwald U., Schmitz H.P., Heinisch J.J. (2008). Dissecting sensor functions in cell wall integrity signaling in *Kluyveromyces lactis*. Fungal Genet. Biol..

[39] Futagami T., Goto M. (2012). Putative cell wall integrity sensor proteins in *Aspergillus nidulans*. Commun. Integr. Biol..

[40] Brown N.A., Dos Reis T.F., Goinski A.B., Savoldi M., Menino J., Almeida M.T., Rodrigues F., Goldman G.H. (2014). The *Aspergillus nidulans* signalling mucin MsbA regulates starvation responses, adhesion and affects cellulase secretion in response to environmental cues. Mol. Microbiol..

[41] Gurgel I.L.d.S., Jorge K.T.d.O.S., Malacco N.L.S.d.O., Souza J.A.M., Rocha M.C., Fernandes M.F., Martins F.R.B., Malavazi I., Teixeira M.M., Soriani F.M. (2019). The *Aspergillus fumigatus* mucin MsbA regulates the cell wall integrity pathway and controls recognition of the fungus by the immune system. mSphere.

[42] Xu L., Wang M., Tang G., Ma Z., Shao W. (2019). The endocytic cargo adaptor complex is required for cell-wall integrity via interacting with the sensor FgWsc2B in *Fusarium graminearum*. Curr. Genet..

[43] Perez-Nadales E., Di Pietro A. (2011). The membrane mucin Msb2 regulates invasive growth and plant infection in *Fusarium oxysporum*. Plant Cell.

[44] Xin C., Xing X., Wang F., Liu J., Ran Z., Chen W., Wang G., Song Z. (2020). MrMid2, encoding a cell wall stress sensor protein, is required for conidium production, stress tolerance, microsclerotium formation and virulence in the entomopathogenic fungus *Metarhizium rileyi*. Fungal Genet. Biol..

[45] Liu W., Zhou X., Li G., Li L., Kong L., Wang C., Zhang H., Xu J.R. (2011). Multiple plant surface signals are sensed by different mechanisms in the rice blast fungus for appressorium formation. PLoS Pathog..

[46] Leroch M., Mueller N., Hinsenkamp I., Hahn M. (2015). The signalling mucin Msb2 regulates surface sensing and host penetration via BMP1 MAP kinase signalling in *Botrytis cinerea*. Mol. Plant Pathol..

[47] Lanver D., Mendoza-Mendoza A., Brachmann A., Kahmann R. (2010). Sho1 and Msb2-related proteins regulate appressorium development in the smut fungus *Ustilago maydis*. Plant Cell.

[48] So Y.S., Jang J., Park G., Xu J., Olszewski M.A., Bahn Y.S. (2018). Sho1 and Msb2 play complementary but distinct roles in stress responses, sexual differentiation, and pathogenicity of *Cryptococcus neoformans*. Front. Microbiol..

[49] Fu C., Iyer P., Herkal A., Abdullah J., Stout A., Free S.J. (2011). Identification and characterization of genes required for cell-to-cell fusion in *Neurospora crassa*. Eukaryot. Cell.

[50] Cullen P.J. (2007). Signaling mucins: The new kids on the MAPK block. Crit. Rev. Eukaryot. Gene Expr..

[51] Vadaie N., Dionne H., Akajagbor D.S., Nickerson S.R., Krysan D.J., Cullen P.J. (2008). Cleavage of the signaling mucin Msb2 by the aspartyl protease Yps1 is required for MAPK activation in yeast. J. Cell Biol..

[52] Rodriguez-Pena J.M., Diez-Muniz S., Bermejo C., Nombela C., Arroyo J. (2013). Activation of the yeast cell wall integrity MAPK pathway by zymolyase depends on protease and glucanase activities and requires the mucin-like protein Hkr1 but not Msb2. FEBS Lett..

[53] Kitamura K., Kaneko T., Yamamoto Y. (1974). Lysis of viable yeast cells by enzymes of *Arthrobacter luteus*: II. Purification and properties of an enzyme, zymolyase, which lyses viable yeast cells. J. Gen. Appl. Microbiol..

[54] Rispail N., Soanes D.M., Ant C., Czajkowski R., Grunler A., Huguet R., Perez-Nadales E., Poli A., Sartorel E., Valiante V. (2009). Comparative genomics of MAP kinase and calcium-calcineurin signalling components in plant and human pathogenic fungi. Fungal Genet. Biol..

[55] Roman E., Cottier F., Ernst J.F., Pla J. (2009). Msb2 signaling mucin controls activation of Cek1 mitogen-activated protein kinase in *Candida albicans*. Eukaryot. Cell.

[56] Wang G., Li G., Zhang S., Jiang C., Qin J., Xu J.R. (2015). Activation of the signalling mucin MoMsb2 and its functional relationship with Cbp1 in *Magnaporthe oryzae*. Environ. Microbiol..

[57] Kamakura T., Yamaguchi S., Saitoh K.-i., Teraoka T., Yamaguchi I. (2002). A novel gene, CBP1, encoding a putative extracellular chitin-binding protein, may play an important role in the hydrophobic surface sensing of *Magnaporthe grisea* during appressorium differentiation. Mol. Plant Microbe Interact..

[58] Wagener J., Striegler K., Wagener N., Latgé J.-P. (2020). α- and β-1,3-Glucan synthesis and remodeling. The Fungal Cell Wall: An Armour and a Weapon for Human Fungal Pathogens.

[59] Samantaray S., Neubauer M., Helmschrott C., Wagener J. (2013). Role of the guanine nucleotide exchange factor Rom2 in cell wall integrity maintenance of *Aspergillus fumigatus*. Eukaryot. Cell.

[60] Kanno T., Takekawa D., Miyakawa Y. (2015). Analysis of the essentiality of ROM2 genes in the pathogenic yeasts *Candida glabrata* and *Candida albicans* using temperature-sensitive mutants. J. Appl. Microbiol..

[61] Beauvais A., Bruneau J.M., Mol P.C., Buitrago M.J., Legrand R., Latgè J.P. (2001). Glucan synthase complex of *Aspergillus fumigatus*. J. Bacteriol..

[62] Dichtl K., Ebel F., Dirr F., Routier F.H., Heesemann J., Wagener J. (2010). Farnesol misplaces tip-localized Rho proteins and inhibits cell wall integrity signalling in *Aspergillus fumigatus*. Mol. Microbiol..

[63] Kwon M.J., Arentshorst M., Roos E.D., van den Hondel C.A., Meyer V., Ram A.F. (2011). Functional characterization of Rho GTPases in *Aspergillus niger* uncovers conserved and diverged roles of Rho proteins within filamentous fungi. Mol. Microbiol..

[64] Guest G.M., Lin X., Momany M. (2004). *Aspergillus nidulans* RhoA is involved in polar growth, branching, and cell wall synthesis. Fungal Genet. Biol..

[65] Ridley A.J. (2006). Rho GTPases and actin dynamics in membrane protrusions and vesicle trafficking. Trends Cell Biol..

[66] Martinez-Rocha A.L., Roncero M.I., Lopez-Ramirez A., Marine M., Guarro J., Martinez-Cadena G., Di Pietro A. (2008). Rho1 has distinct functions in morphogenesis, cell wall biosynthesis and virulence of *Fusarium oxysporum*. Cell. Microbiol..

[67] Pham C.D., Yu Z., Sandrock B., Bolker M., Gold S.E., Perlin M.H. (2009). *Ustilago maydis* Rho1 and 14-3-3 homologues participate in pathways controlling cell separation and cell polarity. Eukaryot. Cell.

[68] Zan X.Y., Zhu H.A., Jiang L.H., Liang Y.Y., Sun W.J., Tao T.L., Cui F.J. (2020). The role of Rho1 gene in the cell wall integrity and polysaccharides biosynthesis of the edible mushroom *Grifola frondosa*. Int. J. Biol. Macromol..

[69] Heinisch J.J., Rodicio R. (2018). Protein kinase C in fungi-more than just cell wall integrity. FEMS Microbiol. Rev..

[70] Schmitz H.P., Jockel J., Block C., Heinisch J.J. (2001). Domain shuffling as a tool for investigation of protein function: Substitution of the cysteine-rich region of Raf kinase and PKC η for that of yeast Pkc1p. J. Mol. Biol..

[71] Schmitz H.-P., Lorberg A., Heinisch J.J. (2002). Regulation of yeast protein kinase C activity by interaction with the small GTPase Rho1p through its amino-terminal HR1 domain. Mol. Microbiol..

[72] Gow N.A.R., Latge J.P., Munro C.A. (2017). The fungal cell wall: Structure, biosynthesis, and function. Microbiol. Spectr..

[73] Paravicini G., Mendoza A., Antonsson B., Cooper M., Losberger C., Payton M.A. (1996). The *Candida albicans* PKC1 gene encodes a protein kinase C homolog necessary for cellular integrity but not dimorphism. Yeast.

[74] Yoshimi A., Miyazawa K., Abe K. (2016). Cell wall structure and biogenesis in *Aspergillus* species. Biosci. Biotechnol. Biochem..

[75] Herrmann M., Sprote P., Brakhage A.A. (2006). Protein kinase C (PkcA) of *Aspergillus nidulans* is involved in penicillin production. Appl. Environ. Microbiol..

[76] Ichinomiya M., Uchida H., Koshi Y., Ohta A., Horiuchi H. (2007). A protein kinase C-encoding gene, *pkcA*, is essential to the viability of the filamentous fungus *Aspergillus nidulans*. Biosci. Biotechnol. Biochem..

[77] Ronen R., Sharon H., Levdansky E., Romano J., Shadkchan Y., Osherov N. (2007). The *Aspergillus nidulans pkcA* gene is involved in polarized growth, morphogenesis and maintenance of cell wall integrity. Curr. Genet..

[78] Teepe A.G., Loprete D.M., He Z., Hoggard T.A., Hill T.W. (2007). The protein kinase C orthologue PkcA plays a role in cell wall integrity and polarized growth in *Aspergillus nidulans*. Fungal Genet. Biol..

[79] Katayama T., Uchida H., Ohta A., Horiuchi H. (2012). Involvement of protein kinase C in the suppression of apoptosis and in polarity establishment in *Aspergillus nidulans* under conditions of heat stress. PLoS ONE.

[80] Katayama T., Ohta A., Horiuchi H. (2015). Protein kinase C regulates the expression of cell wall-related genes in RlmA-dependent and independent manners in *Aspergillus nidulans*. Biosci. Biotechnol. Biochem..

[81] Rocha M.C., Godoy K.F., de Castro P.A., Hori J.I., Bom V.L., Brown N.A., Cunha A.F., Goldman G.H., Malavazi I. (2015). The *Aspergillus fumigatus pkcA^G579R^* mutant is defective in the activation of the cell wall integrity pathway but is dispensable for virulence in a neutropenic mouse infection model. PLoS ONE.

[82] Arpaia G., Cerri F., Baima S., Macino G. (1999). Involvement of protein kinase C in the response of *Neurospora crassa* to blue light. Mol. Gen. Genet..

[83] Franchi L., Fulci V., Macino G. (2005). Protein kinase C modulates light responses in *Neurospora* by regulating the blue light photoreceptor WC-1. Mol. Microbiol..

[84] Penn T.J., Wood M.E., Soanes D.M., Csukai M., Corran A.J., Talbot N.J. (2015). Protein kinase C is essential for viability of the rice blast fungus *Magnaporthe oryzae*. Mol. Microbiol..

[85] Sugahara A., Yoshimi A., Shoji F., Fujioka T., Kawai K., Umeyama H., Komatsu K., Enomoto M., Kuwahara S., Hagiwara D. (2019). Novel antifungal compound Z-705 specifically inhibits protein kinase C of filamentous fungi. Appl. Environ. Microbiol..

[86] Tsuji G., Kenmochi Y., Takano Y., Sweigard J., Farrall L., Furusawa I., Horino O., Kubo Y. (2000). Novel fungal transcriptional activators, Cmr1p of *Colletotrichum lagenarium* and pig1p of *Magnaporthe grisea*, contain Cys2His2 zinc finger and Zn(II)2Cys6 binuclear cluster DNA-binding motifs and regulate transcription of melanin biosynthesis genes in a developmentally specific manner. Mol. Microbiol..

[87] Eliahu N., Igbaria A., Rose M.S., Horwitz B.A., Lev S. (2007). Melanin biosynthesis in the maize pathogen *Cochliobolus heterostrophus* depends on two mitogen-activated protein kinases, Chk1 and Mps1, and the transcription factor Cmr1. Eukaryot. Cell.

[88] Jung U.S., Levin D.E. (1999). Genome-wide analysis of gene expression regulated by the yeast cell wall integrity signalling pathway. Mol. Microbiol..

[89] Martin H., Rodriguez-Pachon J.M., Ruiz C., Nombela C., Molina M. (2000). Regulatory mechanisms for modulation of signaling through the cell integrity Slt2-mediated pathway in *Saccharomyces cerevisiae*. J. Biol. Chem..

[90] de Nobel H., Ruiz C., Martin H., Morris W., Brul S., Molina M., Klis F.M. (2000). Cell wall perturbation in yeast results in dual phosphorylation of the Slt2/Mpk1 MAP kinase and in an Slt2-mediated increase in FKS2-lacZ expression, glucanase resistance and thermotolerance. Microbiology.

[91] Bermejo C., Rodriguez E., Garcia R., Rodriguez-Pena J.M., Rodriguez de la Concepcion M.L., Rivas C., Arias P., Nombela C., Posas F., Arroyo J. (2008). The sequential activation of the yeast HOG and SLT2 pathways is required for cell survival to cell wall stress. Mol. Biol. Cell.

[92] Garcia R., Bermejo C., Grau C., Perez R., Rodriguez-Pena J.M., Francois J., Nombela C., Arroyo J. (2004). The global transcriptional response to transient cell wall damage in *Saccharomyces cerevisiae* and its regulation by the cell integrity signaling pathway. J. Biol. Chem..

[93] Garcia R., Rodriguez-Pena J.M., Bermejo C., Nombela C., Arroyo J. (2009). The high osmotic response and cell wall integrity pathways cooperate to regulate transcriptional responses to zymolyase-induced cell wall stress in *Saccharomyces cerevisiae*. J. Biol. Chem..

[94] Hayes B.M., Anderson M.A., Traven A., van der Weerden N.L., Bleackley M.R. (2014). Activation of stress signalling pathways enhances tolerance of fungi to chemical fungicides and antifungal proteins. Cell. Mol. Life Sci..

[95] Yoshimi A., Fujioka T., Mizutani O., Marui J., Hagiwara D., Abe K. (2015). Mitogen-activated protein kinases MpkA and MpkB independently affect micafungin sensitivity in *Aspergillus nidulans*. Biosci. Biotechnol. Biochem..

[96] Manfiolli A.O., Siqueira F.S., Dos Reis T.F., Van Dijck P., Schrevens S., Hoefgen S., Foge M., Strassburger M., de Assis L.J., Heinekamp T. (2019). Mitogen-activated protein kinase cross-talk interaction modulates the production of melanins in *Aspergillus fumigatus*. mBio.

[97] Jiang C., Zhang X., Liu H., Xu J.R. (2018). Mitogen-activated protein kinase signaling in plant pathogenic fungi. PLoS Pathog..

[98] Izumitsu K., Yoshimi A., Kubo D., Morita A., Saitoh Y., Tanaka C. (2009). The MAPKK kinase ChSte11 regulates sexual/asexual development, melanization, pathogenicity, and adaptation to oxidative stress in *Cochliobolus heterostrophus*. Curr. Genet..

[99] Dichtl K., Samantaray S., Wagener J. (2016). Cell wall integrity signalling in human pathogenic fungi. Cell. Microbiol..

[100] de Oliveira H.C., Rossi S.A., García-Barbazán I., Zaragoza Ó., Trevijano-Contador N. (2021). Cell wall integrity pathway involved in morphogenesis, virulence and antifungal susceptibility in *Cryptococcus neoformans*. J. Fungi.

[101] Chen D.D., Shi L., Yue S.N., Zhang T.J., Wang S.L., Liu Y.N., Ren A., Zhu J., Yu H.S., Zhao M.W. (2019). The Slt2-MAPK pathway is involved in the mechanism by which target of rapamycin regulates cell wall components in *Ganoderma lucidum*. Fungal Genet. Biol..

[102] Lian L., Zhang G., Zhu J., Wang Y., Wang L., Liu R., Shi L., Ren A., Zhao M. (2021). Swi6B, an alternative splicing isoform of Swi6, mediates the cell wall integrity of *Ganoderma lucidum*. Environ. Microbiol..

[103] Denning D.W. (2003). Echinocandin antifungal drugs. Lancet.

[104] Kauffman C.A., Carver P.L. (2008). Update on echinocandin antifungals. Semin. Respir. Crit. Care Med..

[105] Walker L.A., Gow N.A.R., Munro C.A. (2010). Fungal echinocandin resistance. Fungal Genet. Biol..

[106] Osada H. (2019). Special issue: Nucleoside antibiotics, polyoxin and beyond. J. Antibiot.

[107] Endo A., Misato T. (1969). Polyoxin D, a competitive inhibitor of UDP-N-acetylglucosamine: Chitin N-acetylglycosaminyltransferase in *Neurospora crassa*. Biochem. Biophys. Res. Commun..

[108] Endo A., Kakiki K., Misato T. (1970). Mechanism of action of the antifugal agent polyoxin D. J. Bacteriol..

[109] Isono K., Nagatsu J., Kawashima Y., Suzuki S. (1965). Studies on polyoxins, antifungal antibiotics: Part I. Isolation and characterization of polyoxins A and B. Agric. Biol. Chem..

[110] Isono K., Asahi K., Suzuki S. (1969). Polyoxins, antifungal antibiotics. XIII. Structure of polyoxins. J. Am. Chem. Soc..

[111] Serpi M., Ferrari V., Pertusati F. (2016). Nucleoside Derived Antibiotics to Fight Microbial Drug Resistance: New Utilities for an Established Class of Drugs?. J. Med. Chem..

[112] Fiedler H.-P., Kurth R., Langhärig J., Delzer J., Zähner H. (1982). Nikkomycins: Microbial inhibitors of chitin synthase. J. Chem. Technol. Biotechnol..

[113] Wood P.J. (1980). Specificity in the interaction of direct dyes with polysaccharides. Carbohydr. Res..

[114] Ram A.F.J., Klis F.M. (2006). Identification of fungal cell wall mutants using susceptibility assays based on Calcofluor white and Congo red. Nat. Protoc..

[115] Yoshimi A., Sano M., Inaba A., Kokubun Y., Fujioka T., Mizutani O., Hagiwara D., Fujikawa T., Nishimura M., Yano S. (2013). Functional analysis of the α-1,3-glucan synthase genes *agsA* and *agsB* in *Aspergillus nidulans*: AgsB is the major α-1,3-glucan synthase in this fungus. PLoS ONE.

[116] Liu Z., Raj S., van Rhijn N., Fraczek M., Michel J.P., Sismeiro O., Legendre R., Varet H., Fontaine T., Bromley M. (2021). Functional genomic and biochemical analysis reveals pleiotropic effect of congo red on *Aspergillus fumigatus*. mBio.

[117] Lawry S.M., Tebbets B., Kean I., Stewart D., Hetelle J., Klein B.S. (2017). Fludioxonil induces Drk1, a fungal group III hybrid histidine kinase, to dephosphorylate its downstream target, Ypd1. Antimicrob. Agents Chemother..

[118] Brandhorst T.T., Kean I.R.L., Lawry S.M., Wiesner D.L., Klein B.S. (2019). Phenylpyrrole fungicides act on triosephosphate isomerase to induce methylglyoxal stress and alter hybrid histidine kinase activity. Sci. Rep..

[119] Yoshimi A., Hagiwara D., Ono M., Fukuma Y., Midorikawa Y., Furukawa K., Fujioka T., Mizutani O., Sato N., Miyazawa K. (2021). Downregulation of the *ypdA* gene encoding an intermediate of His-Asp phosphorelay signaling in *Aspergillus nidulans* induces the same cellular effects as the phenylpyrrole fungicide fludioxonil. Front. Fungal Biol..

[120] Kojima K., Takano Y., Yoshimi A., Tanaka C., Kikuchi T., Okuno T. (2004). Fungicide activity through activation of a fungal signalling pathway. Mol. Microbiol..

[121] Beattie S.R., Krysan D.J. (2021). A unique dual-readout high-throughput screening assay to identify antifungal compounds with *Aspergillus fumigatus*. mSphere.

[122] Fujikawa T., Kuga Y., Yano S., Yoshimi A., Tachiki T., Abe K., Nishimura M. (2009). Dynamics of cell wall components of *Magnaporthe grisea* during infectious structure development. Mol. Microbiol..

[123] Fujikawa T., Sakaguchi A., Nishizawa Y., Kouzai Y., Minami E., Yano S., Koga H., Meshi T., Nishimura M. (2012). Surface α-1,3-glucan facilitates fungal stealth infection by interfering with innate immunity in plants. PLoS Pathog..

[124] Henry C., Latgè J.P., Beauvais A. (2012). α1,3 Glucans are dispensable in *Aspergillus fumigatus*. Eukaryot. Cell.

[125] Beauvais A., Bozza S., Kniemeyer O., Formosa C., Balloy V., Henry C., Roberson R.W., Dague E., Chignard M., Brakhage A.A. (2013). Deletion of the α-(1,3)-glucan synthase genes induces a restructuring of the conidial cell wall responsible for the avirulence of *Aspergillus fumigatus*. PLoS Pathog..

[126] He X.X., Li S.N., Kaminskyj S.G.W. (2014). Characterization of *Aspergillus nidulans* α-glucan synthesis: Roles for two synthases and two amylases. Mol. Microbiol..

[127] Miyazawa K., Yoshimi A., Kasahara S., Sugahara A., Koizumi A., Yano S., Kimura S., Iwata T., Sano M., Abe K. (2018). Molecular mass and localization of α-1,3-glucan in cell wall control the degree of hyphal aggregation in liquid culture of *Aspergillus nidulans*. Front. Microbiol..

[128] He X., Li S., Kaminskyj S. (2017). An amylase-like protein, AmyD, is the major negative regulator for α-glucan synthesis in *Aspergillus nidulans* during the asexual life cycle. Int. J. Mol. Sci..

[129] Miyazawa K., Yamashita T., Takeuchi A., Kamachi Y., Yoshimi A., Tashiro Y., Koizumi A., Ogata M., Yano S., Kasahara S. (2022). A glycosylphosphatidylinositol-anchored α-amylase encoded by *amyD* contributes to a decrease in the molecular mass of cell wall α-1,3-glucan in *Aspergillus nidulans*. Front. Fungal Biol..

[130] Fontaine T., Beauvais A., Loussert C., Thevenard B., Fulgsang C.C., Ohno N., Clavaud C., Prevost M.C., Latgè J.P. (2010). Cell wall α1-3glucans induce the aggregation of germinating conidia of *Aspergillus fumigatus*. Fungal Genet. Biol..

[131] Abe K., Gomi K., Yoshimi A. (2021). Method for Manuifacturing Useful Substance in Which High-Density Cultured Strain of Filamentous Fungi Is Used. U.S. Patent.

[132] Zhang S., Sato H., Ichinose S., Tanaka M., Miyazawa K., Yoshimi A., Abe K., Shintani T., Gomi K. (2017). Cell wall α-1,3-glucan prevents α-amylase adsorption onto fungal cell in submerged culture of *Aspergillus oryzae*. J. Biosci. Bioeng..

[133] Miyazawa K., Yoshimi A., Zhang S., Sano M., Nakayama M., Gomi K., Abe K. (2016). Increased enzyme production under liquid culture conditions in the industrial fungus *Aspergillus oryzae* by disruption of the genes encoding cell wall α-1,3-glucan synthase. Biosci. Biotechnol. Biochem..

[134] Jeennor S., Anantayanon J., Panchanawaporn S., Chutrakul C., Laoteng K. (2019). Morphologically engineered strain of *Aspergillus oryzae* as a cell chassis for production development of functional lipids. Gene.

[135] Tokashiki J., Hayashi R., Yano S., Watanabe T., Yamada O., Toyama H., Mizutani O. (2019). Influence of α-1,3-glucan synthase gene *agsE* on protoplast formation for transformation of *Aspergillus luchuensis*. J. Biosci. Bioeng..

[136] Miyazawa K., Umeyama T., Hoshino Y., Abe K., Miyazaki Y. (2022). Quantitative monitoring of mycelial growth of *Aspergillus fumigatus* in liquid culture by optical density. Microbiol. Spectr..

[137] Speth C., Rambach G., Lass-Flörl C., Howell P.L., Sheppard D.C. (2019). Galactosaminogalactan (GAG) and its multiple roles in *Aspergillus* pathogenesis. Virulence.

[138] Miyazawa K., Yoshimi A., Sano M., Tabata F., Sugahara A., Kasahara S., Koizumi A., Yano S., Nakajima T., Abe K. (2019). Both galactosaminogalactan and α-1,3-glucan contribute to aggregation of *Aspergillus oryzae* hyphae in liquid culture. Front. Microbiol..

[139] Abe K., Yoshimi A., Miyazawa K., Tabata F., Gomi K., Sano M. (2021). Mutant Filamentous Fungus and Substance Production Method in Which Said Mutant Filamentous Fungus Is Used. U.S. Patent.

[140] Mei L., Wang X., Yin Y., Tang G., Wang C. (2021). Conservative production of galactosaminogalactan in *Metarhizium* is responsible for appressorium mucilage production and topical infection of insect hosts. PLoS Pathog..

[141] Miyazawa K., Yoshimi A., Abe K. (2020). The mechanisms of hyphal pellet formation mediated by polysaccharides, α-1,3-glucan and galactosaminogalactan, in *Aspergillus* species. Fungal Biol. Biotechnol..

[142] Gravelat F.N., Ejzykowicz D.E., Chiang L.Y., Chabot J.C., Urb M., Macdonald K.D., al-Bader N., Filler S.G., Sheppard D.C. (2010). *Aspergillus fumigatus* MedA governs adherence, host cell interactions and virulence. Cell. Microbiol..

[143] Chen Y., Le Mauff F., Wang Y., Lu R., Sheppard D.C., Lu L., Zhang S. (2020). The transcription factor SomA synchronously regulates biofilm formation and cell wall homeostasis in *Aspergillus fumigatus*. mBio.

[144] Lee M.J., Geller A.M., Bamford N.C., Liu H., Gravelat F.N., Snarr B.D., Le Mauff F., Chabot J., Ralph B., Ostapska H. (2016). Deacetylation of fungal exopolysaccharide mediates adhesion and biofilm formation. mBio.

[145] Antecka A., Blatkiewicz M., Bizukojc M., Ledakowicz S. (2016). Morphology engineering of basidiomycetes for improved laccase biosynthesis. Biotechnol. Lett..

[146] Zhang J., Zhang J. (2016). The filamentous fungal pellet and forces driving its formation. Crit. Rev. Biotechnol..

[147] Antecka A., Bizukojc M., Ledakowicz S. (2016). Modern morphological engineering techniques for improving productivity of filamentous fungi in submerged cultures. World J. Microbiol. Biotechnol..

[148] Driouch H., Hansch R., Wucherpfennig T., Krull R., Wittmann C. (2012). Improved enzyme production by bio-pellets of *Aspergillus niger*: Targeted morphology engineering using titanate microparticles. Biotechnol. Bioeng..

[149] Ichikawa H., Miyazawa K., Komeiji K., Susukida S., Zhang S., Muto K., Orita R., Takeuchi A., Kamachi Y., Hitosugi M. (2022). Improved recombinant protein production in *Aspergillus oryzae* lacking both α-1,3-glucan and galactosaminogalactan in batch culture with a lab-scale bioreactor. J. Biosci. Bioeng..

[150] Sakuragawa T., Wakai S., Zhang S., Kawaguchi H., Ogino C., Kondo A. (2021). Accelerated glucose metabolism in hyphae-dispersed *Aspergillus oryzae* is suitable for biological production. J. Biosci. Bioeng..

[151] Cairns T.C., Zheng X.M., Zheng P., Sun J.B., Meyer V. (2019). Moulding the mould: Understanding and reprogramming filamentous fungal growth and morphogenesis for next generation cell factories. Biotechnol. Biofuels.

[152] Yin C., Wang B., He P., Lin Y., Pan L. (2014). Genomic analysis of the aconidial and high-performance protein producer, industrially relevant *Aspergillus niger* SH2 strain. Gene.

[153] Sun X.W., Wu H.F., Zhao G.H., Li Z.M., Wu X.H., Liu H., Zheng Z.M. (2018). Morphological regulation of *Aspergillus niger* to improve citric acid production by *chsC* gene silencing. Bioprocess Biosyst. Eng..

[154] Yin X., Shin H.D., Li J., Du G., Liu L., Chen J. (2017). Comparative genomics and transcriptome analysis of *Aspergillus niger* and metabolic engineering for citrate production. Sci. Rep..

[155] Liu H., Zheng Z., Wang P., Gong G., Wang L., Zhao G. (2013). Morphological changes induced by class III chitin synthase gene silencing could enhance penicillin production of *Penicillium chrysogenum*. Appl. Microbiol. Biotechnol..

[156] Lin L., Sun Z., Li J., Chen Y., Liu Q., Sun W., Tian C. (2018). Disruption of *gul*-*1* decreased the culture viscosity and improved protein secretion in the filamentous fungus *Neurospora crassa*. Microb. Cell Fact..

[157] Fiedler M.R., Lorenz A., Nitsche B.M., van den Hondel C.A., Ram A.F., Meyer V. (2014). The capacity of *Aspergillus niger* to sense and respond to cell wall stress requires at least three transcription factors: RlmA, MsnA and CrzA. Fungal Biol. Biotechnol..

